# Conserved Noncoding Sequences Boost ADR1 and SP1 Regulated Human Swiprosin-1 Promoter Activity

**DOI:** 10.1038/s41598-018-34802-z

**Published:** 2018-11-07

**Authors:** Ramesh P. Thylur, Sung Yong Ahn, Eunhea Jung, Chang-Duk Jun, Young-Min Hyun

**Affiliations:** 1 School of Life Sciences, Gwangju Institute of Science and Technology (GIST), Gwangju, 61005 Korea; 2 Department of Anatomy, Yonsei University College of Medicine, Seoul, 03722 Korea; 3 Brain Korea 21 Plus Project for Medical Science, Yonsei University College of Medicine, Seoul, 03722 Korea; 4 Present Address: Department of Biochemistry and Molecular Biology, The Pennsylvania State University College of Medicine, Hershey, Pennsylvania 17033 USA; 5 Immune Synapse and Cell Therapy Resesarch Center, Gwangju Institute of Science and Technology (GIST), Gwangju, 61005 Korea

## Abstract

Swiprosin-1 is expressed in various types of cells or tissues of different species. To investigate the mechanisms underlying Swiprosin-1 expression pattern, we analyzed the promoter activity of 2-kilobase genomic sequences located at 5′ flanking region of the Swiprosin-1 gene. The −2000/+41 bp of 5′ flanking untranslated promoter region of Swiprosin-1 gene was constitutively transactivated without significant effect of PMA, A23187, or PMA/A23187 stimulation in Jurkat T cells. Further, we identified 5′ deletant of proximal promoter region (−100/+41 to −70/+41) plays a pivotal role in activating the Swiprosin-1 gene in Jurkat T cells. Our studies also verified that ADR1 and Sp1 transcription factors were located between −70 and -100 locus of 5′ flanking proximal promoter region, which is critical for the Swiprosin-1 promoter activity. ADR1 and Sp1 were shown to bind the regions of −82, −79, −76, −73 and −70 and; −79, −78 and −77, respectively, within the proximal promoter region of Swiprosin-1. Finally conserved noncoding sequences (CNS) -1, -2 and -3 were located between the exon 1 and exon 2 of Swiprosin-1 gene and synergistically transactivated the Swiprosin-1 promoter. In summary, Swiprosin-1 was constitutively expressed in Jurkat T cells by the coordinate action of ADR1 and SP1 transcription factors at the transcriptional level and CNS further boost the proximal region of Swiprosin-1 promoter activity. Our findings provide novel insights that the transcriptional regulation of Swiprosin-1 by targeting ADR1 and Sp1 binding sites may be helpful in exploring novel therapeutic strategies for advanced immune or other disorders.

## Introduction

Swiprosin-1 is expressed across different types of cell and tissue in various species at variable levels^1^. The Swiprosin-1 expression is more abundant in pathological conditions like inflammation (acute- passive cutaneous anaphylaxis and chronic- atopic dermatitis), sepsis, neurodegeneration, dementia, schizophrenia, diabetic nephropathy, and cancer, as well as in *Enterococcus*.*faecalis*-infected male fertility patients^2–10^. However, the pattern of Swiprosin-1 expression is regulated by various signaling pathways such as PKC-βI/η in HMC-1 mast cells, PKC-θ and NF-κB in Jurkat T cells, BCR in WEHI231 B cells, RANK-L in osteoclast-like cells and EGF in melanoma B16F10 cells, and PKC-β in glomerular endothelial cells^2,6,7,11,12^.

Furthermore, Swiprosin-1 exhibits diverse biological functions^1^ like amplifying cytokines, chemokines, and histamine release, and acts as a positive regulator of NF-κB activation in mast cells^2,13^. Also, Swiprosin-1 acts as a positive regulator for Syk activity, a negative regulator of NF-κB activation, and induces apoptosis in B cells^12,14^. Swiprosin-1 induces phagocytic activity in differentiated macrophages, like the hemocytes of drosophila^15^, and also regulates the BCR-elicited Ca^2+^ flux^16^, cancer invasion, and metastasis in melanoma B16F10 cells^15^. In addition, Swiprosin-1 contributes to lamellipodia formation, cell migration, and regulation of membrane dynamics^17^. Swiprosin-1 regulates neuron axonal transport^4^, but it acts as a negative regulator of germinal center-dependent humoral type 2 immunity^18^.

Swiprosin-1 deficiency reduces mitochondria-dependent podocyte apoptosis, induced by hyperglycemia or high glucose, via p38 MAPK signaling pathway in the early stage of diabetic nephropathy^9^. Swiprosin-1 plays a critical role in the macrophage immune response to LPS- and cecal ligation and puncture-induced sepsis, as well as in the production of inflammatory cytokines like IL-1b, IL-6, TNF-a, IL-10, and IFN-g. In addition, Swiprosin-1 activates the JAK2/STAT1/STAT3 signaling pathway by regulating IFN-γR expression in macrophages, and also contributes to the LPS-stimulated macrophage migration^19,20^. A change in intracellular calcium concentration decreases the Swiprosin-1 expression during cardiac de- and re-differentiation. Also, Swiprosin-1 desensitizes β-adrenergic receptors in the cultured adult ventricular rat cardiomyocytes, suggesting that it may play a key role in cardiac remodeling^21,22^. Swiprosin-1 expression in vestibular nuclei determines the susceptibility of mice to motion sickness^23^.

To explore the mechanisms of Swiprosin-1 in this vein, our research aimed to dissect the critical region of molecules for the transcriptional activity of proximal promoter region of swiprosin-1 gene. In the present study, first time we identified the −2000/+41 bp of 5′ flanking region of Swiprosin-1 gene promoter is constitutively transactivated without showing any significant response to the PMA or A23187 or PMA/A23187 stimulation in the Jurkat T cells. Further −100/+41 bp to −70/+41 bp of proximal promoter region of 5′ flanking Swiprosin-1 gene is critical core region whose transcriptional activity is regulated by two ADR1 and two Sp1 binding sites between −82/+41 bp to −70/+41 bp of 5′ flanking region of Swiprosin-1 gene promoter. Electrophoretic mobility shift assay (EMSA) and chromatin immunoprecipitation assay (ChIP) demonstrated that both ADR1 and Sp1 are the functional transcription activators for Swiprosin-1 gene. In addition, Swiprosin-1 proximal promoter region is synergistically transactivated by (conserved noncoding sequences) CNS -1, -2 and -3, which are located between the exon-1 and exon-2. Taken together, our results highlighted that ADR1 and SP1 transcription factors are pivotal in transactivating the Swiprosin-1 gene in Jurkat T cells constitutively and also its transcriptional activity is further boosted by CNS. Therapeutically targeting ADR1 and SP1 binding sites at Swiprosin-1 promoter region may could regulate the inflammatory or other diseases.

## Results

### Identification of the transcription start site and promoter region of Swiprosin-1

**Figure 1:**
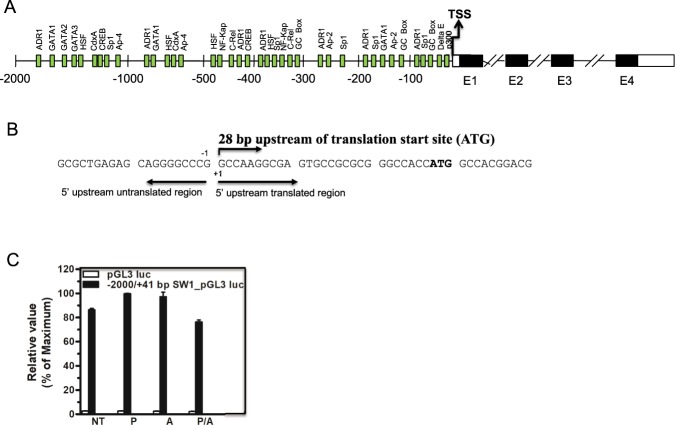
Human Swiprosin-1 promoter activity. (**A**) Schematic diagram of human Swiprosin-1 gene and prediction of important transcription factors binding sites on the 5′ untranslated Swiprosin-1 promoter region by TRANSFAC database. (**B**) Analyzing the DNA sequence (GenBank accession no. NC000001) revealed a major TSS assigned to the G nucleotide 28 bp upstream of the translation start site (ATG). (**C**) Jurkat T cells were transfected with the pGL3 luciferase reporter construct containing the full length −2000/+41 Swiprosin-1 promoters only and basic pGL3 luc as a control. After 36 h of transfection, cells were stimulated with PMA or A23187 and PMA/A23187 for 6 h, and then harvested for assay. The relative luciferase activity (RLA) was expressed as fold difference relative to the unstimulated mock sample. Error bars indicate ± SD. Data are representative of three independent experiments.

To identify the transcription start site (TSS) at the human Swiprosin-1 gene locus, we employed a comparative genomic sequence analysis of rVISTA 2.0 and TRANSFAC database. For this, we isolated the genomic DNA from Jurkat T cell. We then amplified it with the gene specific primer, which corresponds to exon 1 region of Swiprosin-1 gene (Fig. 1A). The PCR product was sequenced and, to determine the location of the TSS, alignment of the sequenced PCR products to the human genome was performed along with GenBank (GenBank accession no. NC_000001 and region, 15607028…15609069). TSS was aligned with 28 bp upstream of translation start site, ATG (Fig. 1B). Next to the promoter analysis of the Swiprosin-1 gene, our previous studies revealed that the increased level of Swiprosin-1 mRNA in HMC-1 and Jurkat T cells with a stimulation of PMA or A23187 or PMA/A23187^2,11^. This result suggests that the Swiprosin-1 gene is controlled at the level of transcription. To study the mechanism of transcription of the Swiprosin-1 gene, the untranslated 5′ upstream promoter (−2 kb) as well as 28 bp of 5′ translated region and 13 bp of CDS (coding sequence) of exon1 (total −2000/+41 bp) were cloned from human Jurkat T cell genomic DNA into the pGL3 reporter plasmid, then transfected into the Jurkat T cells for 36 h and followed by above stimuli for 6 h. The luciferase reporter assay suggested that −2000/+41 bp promoter region of Swiprosin-1 gene showed very marginal level of increased activity to the PMA and A23187 but not to the PMA/A23187 stimuli with respect to the unstimulated one (Fig. 1C). Therefore, we supposed that the promoter region of Swiprosin-1 gene is constitutively activated both in Jurkat T cells (Fig. 1C) and HMC-1 cells (Data not shown).

### Promoter analysis of the Swiprosin-1 gene

**Figure 2:**
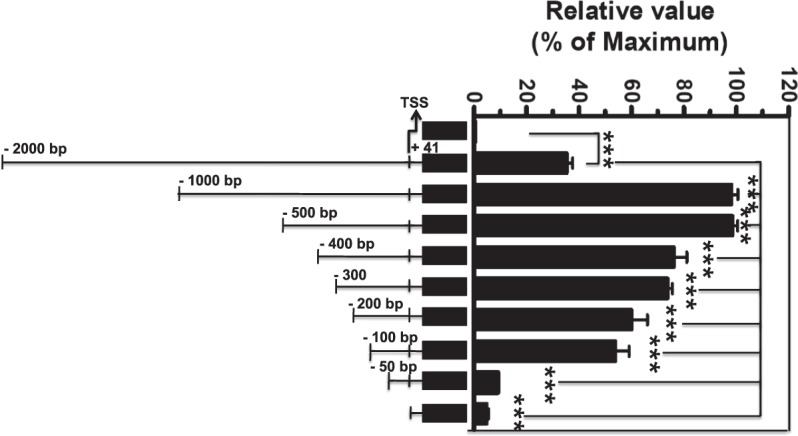
5′ deletion mutants of the human Swiprosin-1 promoter activity. Jurkat T cells were transfected with the control pGL3 luc vector (mock) and the Swiprosin-1 luciferase reporter constructs. Schematic representation of the genomic position and size of the deletion constructs of the 5′ region of the human Swiprosin-1 gene are shown. After 36 h post transfection, cells were harvested for assay. The relative luciferase activity (RLA) was expressed as fold difference relative to the basic pGL3 luc sample. Error bars indicate ± SD. Data are representative of two independent experiments. ***p, 0.001.

To dissect the role of promoter region in Swiprosin-1 gene activity, we produced a series of 5′ deletions of upstream Swiprosin-1 gene promoter by PCR cloning into to the pGL3 reporter plasmid to determine the activity of the promoter fragment. Each Swiprosin-1 reporter plasmid was then tested for transcriptional activity in Jurkat T cells by Amaxa’s Nucleofector transfection. Cell lysates were prepared after transfection and then the level of luciferase activity was measured, as described in *Materials and Methods*. Using luciferase assays, we found that the 5′ upstream untranslated −100/+41 bp promoter region of Swiprosin-1 gene was pivotal for positively regulating this activity (Fig. 2).

### Elements regulate the proximal promoter region of Swiprosin-1 gene

**Figure 3:**
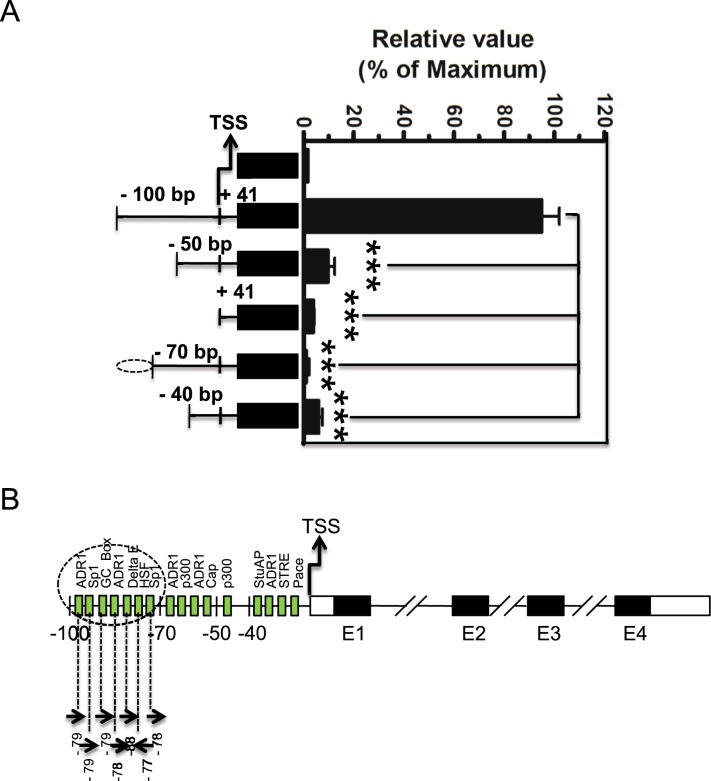
Identification of regulatory elements on the proximal region of human Swiprosin-1 promoter activity. (**A**) Schematic representation of the genomic position and size of the deletion constructs of the 5′ region of the −100/+41 bp human Swiprosin-1 gene are shown. All these deletions of Swiprosin-1 luciferase reporter construct and control basic pGL3 luc vector were transfected into the Jurkat T cells for 36 h and then cells were processed for assay. The relative luciferase activity (RLA) was expressed as fold difference relative to the basic pGL3 luc sample. Error bars indicate ± SD. Data are representative of three independent experiments. ***p, 0.001. (**B**) Schematic diagram of predicted potential binding sites for the transcription factors within the region from −100/+41 bp to −70/+41 bp proximal promoter region of Swiprosin-1 gene analyzed by a comparative genomic sequence of rVISTA 2.0 and TRANSFAC database.

To further investigate the role of proximal promoter region in the −100/+41 bp Swiprosin-1 gene, we performed further 5′ deletions of upstream −100/+41 bp region of Swiprosin-1 gene promoter activity by luciferase assays. These experiments demonstrated that −50/+41 bp, +41 bp, −70/+41 bp, and −40/+41 bp of 5′ deletions significantly decreased the promoter activity, compared to the −100/+41 bp 5′ deletion. These results also suggested that the 5′ deletion of the upstream untranslated region between the −100/+41 bp and −70/+41 bp is the critical proximal promoter region of Swiprosin-1 gene activity (Fig. 3A). Therefore, we confirmed that several potential regulatory elements are located between the −100/+41 bp and −70/+41 bp of 5′ untranslated proximal promoter region of Swiprosin-1 gene, based on analysis using a comparative genomic sequence of rVISTA 2.0 and TRANSFAC database (Fig. 3B).

### ADR1 and Sp1 regulate the proximal promoter region of Swiprosin-1 gene, which is located between −100/+41 bp and −70/+41 bp

**Figure 4:**
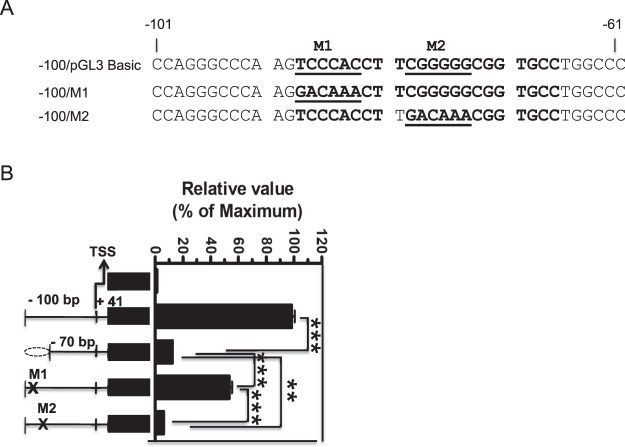
ADR1 and Sp1 regulates the proximal region of Swiprosin-1 promoter activity. (**A**) Schematic representation of nucleotides was mutated as M1 and M2 (bolded and underlined) between −83 to −88 and −74 to −79 biding sites of ADR1 and Sp1 on −100/+41 bp to −70/+41 bp proximal region of Swiprosin-1 gene. (**B**) Mutants M1 and M2 constructs were transfected into the Jurkat T cells for 36 h and cells were processed for assay. The relative luciferase activity (RLA) was expressed as fold difference relative to the basic pGL3 luc sample. Error bars indicate ± SD. Data are representative of three independent experiments. **p, 0.005, ***p, 0.001.

To find out whether ADR1 and Sp1 transcription factors or elements regulate the proximal promoter region of Swiprosin-1 gene activity, we employed over-lapping PCR and mutated as M1 and M2 clones at the ADR1 and Sp1 binding sites, respectively, located between the −100/+41 bp and −70/+41 bp of proximal promoter Swiprosin-1 gene locus (Fig. 4A). The luciferase reporter assay revealed that both M1 and M2 mutants showed decreased promoter activity compared to the −100/+41 bp proximal promoter region (Fig. 4B). Furthermore, M1 mutant showed significantly increased promoter activity in contrast to −70/+41 bp proximal promoter region. However, M2 mutant showed very significant decreased promoter activity similar to that of basic pGL3 reporter as compared with the −70/+41 bp proximal promoter region of Swiprosin-1 gene (Fig. 4B). Taken together, our results conclude that both ADR1 and Sp1 crucially modulate the proximal promoter region of Swiprosin-1 gene.

### *In vitro* and *In vivo* binding assays of ADR1 and Sp1 to the proximal promoter region of Swiprosin-1 gene

**Figure 5:**
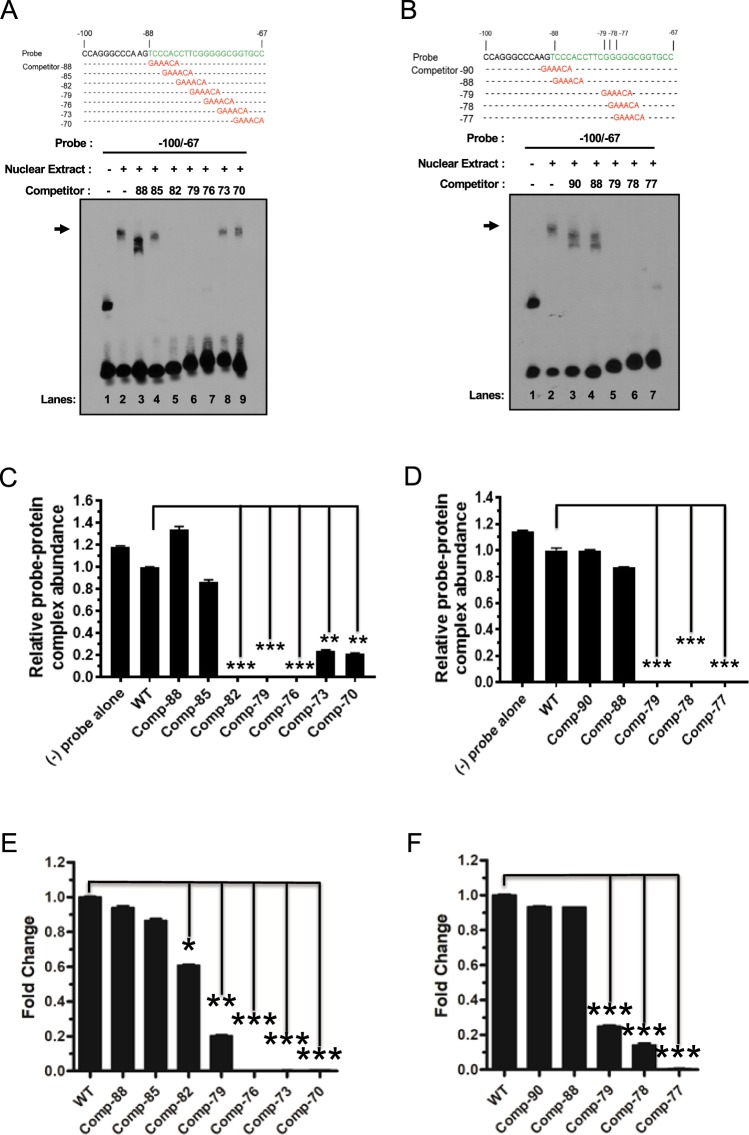
*In vitro* and *in vivo* binding of ADR1 and Sp1 to the proximal promoter region of Swiprosin-1 gene. (**A**,**B**) EMSA with the indicated biotin labeled probes and competitor oligonucleotides were added where indicated. Nuclear extracts were obtained from Jurkat T cells. Arrows indicate position of specific DNA-nuclear protein complexes. (**C**,**D**) The band intensity on each blot was normalized to the band of free probe, and the relative probe-protein complex abundance is shown as the ratio to that in wild type. Error bars indicate ± SD. Data are representative of three independent experiments. **p, 0.005, ***p, 0.001 compared to wild type by Mann-Whitney test. (**E**,**F**) ChIP assay was performed using Jurkat T cells, and the levels of precipitated DNA were measured by RT-qPCR with wild type and competitor primers specific for Swiprosin-1 promoter as indicated. Error bars indicate ± SD. Data are representative of three independent experiments. *p, 0.05, **p, 0.01, ***p, 0.005 compared to wild type by Mann-Whitney test.

To verify whether ADR1 and Sp1 bind to the proximal promoter region −100/+41 to −70/+41 bp of Swiprosin-1 *in vitro*, we conducted EMSA using nuclear extracts prepared from Jurkat T cells with biotin labeled probes in the presence of antibody against ADR1 and Sp1, respectively. Wild type probe alone was used as a negative control (Fig. 5A,B, lane 1). Indeed, wild type ADR1 oligonucleotide probe binding to the Swiprosin-1 promoter sequence was observed (Fig. 5A, lane 2, upper band marked by arrow). However, in the presence of competitor oligonucleotide probes −88 and −85, complex formation was observed, which implies that the competition of ADR1 binding was not successful (Fig. 5A,C, lanes 3 and 4). On the other hand, in the presence of competitor oligonucleotide probes −73 (p < 0.005) and −70 (p < 0.005), the level of ADR1 binding was significantly decreased compared to wild type, despite complex formation (Fig. 5A and C, lane 8 and 9). In contrast, in the presence of competitor oligonucleotide probes −82 (p < 0.001), −79 (p < 0.001), and −76 (p < 0.001), complex formation was not observed, indicating that the level of ADR1 binding was significantly decreased (Fig. 5A and C, lanes 5, 6 and 7). In the same manner, wild type Sp1 oligonucleotide probe binding to the Swiprosin-1 promoter sequence was observed (Fig. 5B, lane 2, upper band marked by arrow). However, in the presence of competitor oligonucleotide probes −90 and −88, complex formation was still observed, which demonstrated that the competition of Sp1 binding was not successful (Fig. 5B and D, lanes 3 and 4). In addition, the presence of competitor oligonucleotide probes −79 (p < 0.001), −78 (p < 0.001), and −77 (p < 0.001) obviously diminished complex formation by verifying marked decrease of Sp1 binding (Fig. 5B and D, lanes 5, 6, and 7).

To further investigate whether these ADR1 and Sp1 factors bind to Swiprosin-1 promoter region *in vivo*, ChIP assay using real-time quantitative PCR was performed in Jurkat T cells (Fig. 5E and F). Binding of both endogenous ADR1 and Sp1 to the Swiprosin-1 promoter locus was highly enriched in Jurkat T cells. Similar to the result of EMSA of ADR1, in the presence of competitor oligonucleotide primers −88 and −85, Swiprosin-1 mRNA expression did not decrease, whereas another competitor oligonucleotide primer −82 (p < 0.05) significantly decreased Swiprosin-1 mRNA expression. In addition, in the presence of competitor oligonucleotide primers −79 (p < 0.01), −76 (p < 0.005), −73 (p < 0.005), and −70 (p < 0.005) significantly decreases the Swiprosin-1 mRNA expression (Fig. 5E). In accordance with the result of EMSA of Sp1, in the presence of competitor oligonucleotide primers −90 and −88, Swiprosin-1 mRNA expression level was similar compared to that of the control, whereas the presence of competitor oligonucleotide primers −79 (p < 0.005), −78 (p < 0.005), and −77 (p < 0.005) markedly decreased Swiprosin-1 mRNA expression (Fig. 5F). Collectively our results indicate that both ADR1 and Sp1 sites are physically associated with the Swiprosin-1 promoter region.

### Conserved noncoding sequences significantly upregulate the proximal promoter region of Swiprosin-1 gene

**Figure 6:**
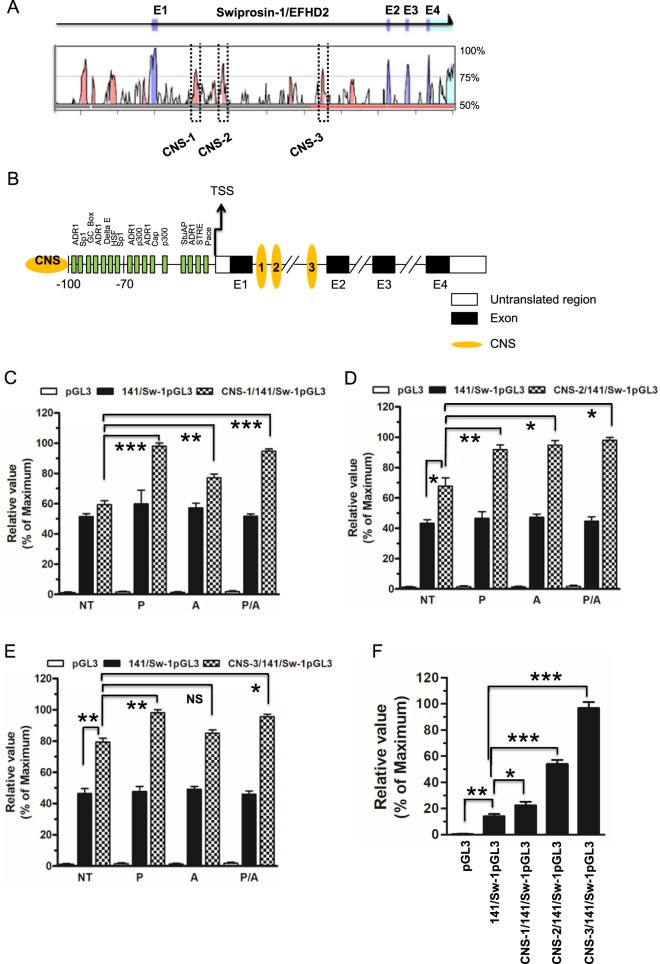
Conserved noncoding sequences amplify the transactivation of proximal promoter region of Swiprosin-1. (**A**) ECR browser analysis of the human Swiprosin-1 loci is shown. The human genomic sequence is used as the base sequence on the x-axis. Schematic representation of the genomic positions of exons (**E**) and boxed region indicates the conserved noncoding sequences (CNS) in the Swiprosin-1 loci of human has shown. (**B**) Schematic diagram of CNS regions is inserted into the 5′ upstream of proximal promoter region (−100/+41 bp) of Swiprosin-1 gene. (**C**–**E**) Jurkat T cells were transfected with the pGL3 luciferase reporter construct containing CNS regions with or without −100/+41 bp proximal promoter region of Swiprosin-1 gene and basic pGL3 luc as a control. After 36 h of transfection, cells were stimulated with PMA or A23187 and PMA/A23187 for 6 h, and then harvested for assay. (**F**) Jurkat T cells were transfected with the pGL3 luciferase reporter construct containing CNS regions with or without −100/+41 bp proximal promoter region of Swiprosin-1 gene and basic pGL3 luc as a control. After 36 h of transfection, without any above treatments cells were harvested for assay. The relative luciferase activity (RLA) was expressed as fold difference relative to the unstimulated mock sample. Error bars indicate ± SD. Data are representative of two independent experiments. *p, 0.05, **p, 0.005, ***p, 0.001.

To investigate whether the molecular mechanisms underlying the conserved noncoding sequences (CNS) depend on the regulation of proximal promoter region of Swiprosin-1 gene transactivation, we employed a comparative genomic sequence of rVISTA 2.0 ECR browser analysis for the schematic representation of the genomic positions of exons (E) and boxed region indicates the conserved noncoding sequences (CNS) in the Swiprosin-1 loci of human (Fig. 6A). For this study, we cloned CNS -1, -2, and -3 at 5′ of the luciferase reporter gene construct driven by a −100/+41 bp fragment encompassing the proximal promoter region for human Swiprosin-1 (Fig. 6B). Transiently transfected Jurkat T cells of CNS-1/-141Sw-1, CNS-2/-141Sw-1 and CNS-3/-141Sw-1 or −100/+41Sw-1 alone or empty pGL3 reporter constructs were treated with PMA or A23187 or PMA/A23187 for 6 h. Through luciferase reporter assay, we found that CNS-1 increased ~1.7, ~1.4 and ~1.7 folds (Fig. 6C), whereas CNS-2 increased ~1.3, ~1.33 and ~1.35 folds (Fig. 6D), and CNS-3 increased ~1.2, ~1.0 and ~1.2 folds (Fig. 6E) of -141/Sw-1 proximal promoter gene transcription respectively upon above treatments, when compared to the un-treated one as a control.

Further, CNS-1, -2 and -3 of -141/Sw-1 proximal promoter activity were ~1.5 to ~2 fold increased in contrast to the -141/Sw-1 proximal promoter (without CNS regions conjugated) upon above treatments (Fig. 6C–E). However, CNS -2 and -3, but not CNS-1, were constitutively transactivated (~2 fold) at the proximal promoter region of -141/Sw-1 gene in the absence of above treatments (Fig. 6C–E). The proximal promoter region of -141/Sw-1 gene (without CNS regions conjugated) was not affected by any of the above treatments compared with un-treated as a control (Fig. 6C–E). Notably, CNS-1, -2 and -3 significantly increased the proximal promoter region of -141/Sw-1 gene transcription activation ~1.5, ~3.5 and ~6.4 folds respectively in the absence of any above treatments (Fig. 6F). Overall our results suggest that proximal promoter region of -141/Sw-1gene transcription activation is constitute but this activation is further amplified by CNS with or without treatments and, more importantly, CNS-2 and -3 constitutively transactivate the proximal promoter region of -141/Sw-1 gene significantly (Fig. 6F).

## Discussion

The main purpose of this study was to elucidate the molecular mechanism of Swiprosin-1 gene regulation in T cells in the health and disease status. In the present study, we identified the TSS and also defined that the region from −100/+41 to −70/+41 was the functional core promoter region for Swiprosin-1 gene. In addition, ADR1 and Sp1 were identified as transactivators of Swiprosin-1 gene. This conclusion was supported by the following lines of evidence: (i) a series of deletions revealed that the region spanning −100/+41 to −70/+41 possessed an essential transcriptional activity; (ii) ADR1 and Sp1 binding sites region were mapped to the promoter region of the Swiprosin-1 gene, and mutation of these sites caused a dramatic decrease in the Swiprosin-1 promoter activity; (iii) ADR1 and Sp1 were directly interacted with the Swiprosin-1 promoter *in vitro* and *in vivo*. Taken together, these findings show that ADR1 and Sp1 may serve as an important transcription factors for Swiprosin-1.

The expression and biological function of Swiprosin-1 gene have been reported to be elevated and significantly correlated with the clinical outcome in health and various diseases^1,2,6–13,24^. However, the precise mechanisms underlying Swiprosin-1 gene regulation was still elusive. Here, by performing bioinformatics analysis of 5′ upstream region of Swiprosin-1gene locus, the −2000 bp full length promoter region showed very marginal level increase of transcription activity up on PMA or A23187 but not to the PMA/A23187 stimulation (Fig. 1C) as compared with the unstimulated one. We speculated that −2000 bp full length promoter region of Swiprosin-1gene was transactivated constitutively as compared to the basic pGL3 reporter gene. This result was correlated with that the Swiprosin-1gene expression was not upregulated in mouse B cells upon anti-IgM F(ab)2/IL-4, LPS or anti-CD40/IL-4^12^. However, our previous studies and others reported that endogenous Swiprosin-1gene expression was specifically upregulated in human mast and T cells up on PMA or A23187 or PMA/A23187 stimulation and also in malignant melanoma cells upon EGFR stimulation^2,6,7,9,11^. Swiprosin-1 gene expression was very broad and also its expression level was tissue or cell type specific of various organisms^1,9,19,20^. Therefore, we speculate that conserved noncoding sequences may be transactivate the promoter region of Swiprosin-1 gene upon stimulation.

Further, a serious of 5′ deletion of Swiprosin-1 gene promoter studies revealed that −100/+41 regions (Fig. 2) was pivotal for constitutively transactivated the Swiprosin-1gene promoter. Next, by a comparative genomic sequence of rVISTA 2.0 and TRANSFAC database analysis^25^ and our further 5′ deletion of Swiprosin-1 gene promoter transactivation, we suggested that region between the −100/+41 bp and −70/+41 bp is the critical proximal promoter region of Swiprosin-1 gene activity (Fig. 3A) and also several potential regulatory elements are located between the above proximal promoter region (Fig. 3B). Over lapping PCR studies showed that ADR1 and Sp1 elements regulate the transactivation of proximal promoter region of Swiprosin-1 gene. Mutating ADR1 and Sp1 sites as M1 and M2 on the proximal promoter region of Swiprosin-1 gene locus showed significantly decreased promoter activity as 50% and 90% respectively (Fig. 4A,B). Notably, many known transcription factors may serve as a central mediator for transactivating the various immune responsive genes. For examples, ADR1 transactivate the repression of the glucose-repressible *ADH2* gene in *Saccharomyces*.*cerevisiae*^26^. Sp1 and c-Myc regulate transcription of BMI1 in nasopharyngeal carcinoma^27^ and NFAT regulate CTLA-4 gene in human lymphocytes^28^. Both NFAT1 and Jun B regulate IL-31 gene expression in CD4+ T cells in health and disease^25^. NF-κB controls the transcription of over 150 target genes^29^. Similarly, our overall results suggest that both ADR1 and Sp1 regulate the transactivation of proximal promoter region of Swiprosin-1 gene.

To further confirm whether ADR1 and Sp1 bind to the proximal promoter region −100/+41 to −70/+41 bp of Swiprosin-1 *in vitro* and *in vivo*, both EMSA and Chip assays were performed with a specific antibody against ADR1 or Sp1. Interestingly, these findings suggested that both ADR1 and Sp1 are essential transcription factors for Swiprosin-1 promoter. The specificity of the binding sites and the factors binding there were shown by competition studies by using consensus sequences of the transcription factor binding sites and the Abs to both ADR1 and Sp1 in EMSA experiments (Fig. 5A–D). Mutations in the ADR1 and Sp1 binding sites significantly prevented formation of the protein/DNA complexes (Fig. 5A–D) and also markedly decreased the Swiprosin-1 mRNA expression (Fig. 5E,F). In addition, these results in EMSA and ChIP assays were in accordance with those in the mutated ADR1 and Sp1 bind sites, as M1 and M2 on the proximal promoter region of the Swiprosin-1 gene. These results imply that ADR1 and Sp1 are associated with endogenous Swiprosin-1 expression in human Jurkat T cells. Therefore, we suggest a predominant role of ADR1 and Sp1 in transactivating the Swiprosin-1 gene.

A breakthrough came as a result of comparative genomics analyses to identify noncoding regions highly conserved (>70% identity) between humans and evolutionarily distant mammalian species^30,31^. To find out the molecular mechanisms underlying the conserved noncoding sequences (CNS) dependent regulation of proximal promoter region of Swiprosin-1 gene transcription, we found CNS-1, -2 and -3 were located between the first and second exon of Swiprosin-1 gene (Fig. 6A) by comparative genomic analysis of web-based alignment software VISTA browser 2.0. Here, our results showed that CNS-1/-141Sw-1, CNS-2/-141Sw-1, and CNS-3/-141Sw-1 increased the transactivation of proximal promoter region of Swiprosin-1 gene as ~1.4 to ~1.7, ~1.3 and ~1.2 folds respectively up on PMA or A23187 or PMA/A23187 treatment for 6 h as compared with the untreated control (Fig. 6C–E). Taken together, our present results correlated to the transcription (mRNA) of Swiprosin-1 gene was upregulated upon treatments, which were previously reported in mast cells, T cells, malignant melanoma cells^1^, macrophage, and glomerular endothelial cells^6,9,20^. More importantly, we notified that all CNS-1, -2 and -3 upregulate the transactivation of −141 bp proximal promoter region of Swiprosin-1 compared with the -141/Sw-1 alone (without conjugated with CNS) even in the absence of above treatments, and CNS-3 was most predominant among them (Fig. 6F). This phenomenon was correlated to that endogenous Swiprosin-1 gene constitutively expressed in mouse B cells, but it was not upregulated upon anti-IgM F(ab)2/IL-4, LPS or anti-CD40/IL-4^12^. Here we would argument that endogenous Swiprosin-1 gene expression and also its upregulation upon stimuli depend on the cell or tissue type specific of various organisms and diverse pathophysiological conditions. Further underlying mechanism study would be required to dissect the regulatory elements of conserved noncoding sequences and its binding sites for transactivating the proximal promoter region of Swiprosin-1 gene.

In conclusion, first time we have identified the −100/+41 to −70/+41 bp core promoter regions of Swiprosin-1 gene, and showed ADR1 and Sp1 are important transcription factors that directly bind to and transactivate the Swiprosin-1 promoter region. In addition, CNS-1, -2 and -3 but CNS-3 prominently transactivate the proximal promoter region of Swiprosin-1. Our findings provide us novel insights into the transcriptional regulation of Swiprosin-1, and targeting its ADR1 and Sp1 binding sites may be helpful in the exploration of novel therapeutic strategies for advanced immune or other disorders.

## Methods

### Cells

Jurkat T (TIB-152; ATCC) cells were maintained in RPMI 1640 medium supplemented with 10% heat-inactivated fetal bovine serum (FBS), penicillin G (100 IU/ml) and streptomycin (100 μg/ml) were all from Gibco/Invitrogen.

### Plasmid construction

The deletion constructs were generated by cloning the genomic sequences upstream of the first coding exon of the Swiprosin-1gene into the pGL3 reporter vector digested by appropriate restriction enzymes. Different combinations of forward primers containing Sac I restriction enzyme site and same reverse primer containing Hind III or Bam HI restriction enzyme site in the 28 region of the transcription start site of the human Swiprosin-1 from the genomic DNA of Jurkat T cells were used to obtain the deletion constructs and primer sequences as follows: forward, 2041 bp, 5′-AAA GAG CTC ACC CTC CCT CCT AAG AGC TGT GTC-3′; 1041 bp, 5′-AAA GAG CTC CAG TTT CGT CTG TCC AAA TTG GAG-3′; 541 bp, 5′-AAA GAG CTC ATT GAG AAC GCC AGT GTT TAA CAG-3′; 441 bp, 5′-AAA GAG CTC AGG GGG TGG GGC GGC GCC CCC CCT-3; 341 bp, 5′-AAA GAG CTC CCG GCC CCC AGC AGG A CC CCT CCC-3′; 241 bp, 5′-AAA GAG CTC GCG GGC GGG GCG CGC AGA GTA CAC-3′; 141 bp, 5′-AAA GAG CTC GCC AGG GCC CAA GTC CCA CCT TCG-3′; 91 bp, 5′-AAA GAG CTC AGG AAG AGG AAG AGC GCG GCC GGC-3′; 41 bp, 5′-AAA GAG CTC GCC AAG GCG AGT GCC GCG CGG GCC-3′; 111 bp, 5′-AAA GAG CTC TGC CTG GCC CGG GGA GTG TCA GGA-3′; 81 bp, 5′-AAA GAG CTC AGA GCG CGG CCG GCG GCG CTG CGC-3′; reverse, 2041 bp, 5′-AAA AAG CTT CGT CCG TGG CCA TGG TGG CCC GCG-3′; reverse, 2000 bp, 5′-AAA GGA TCC TTA TCG ATT TTA CCA CAT TTG TAG-3′. The sequences of all the PCR derived constructs were confirmed by DNA sequencing.

### Over-lapping PCR

The binding sites for ADR1 or Sp1 between the −101 and −61 region of Swiprosin-1 promoter locus were subjected to the over**-**lapping PCR and cloned into the pGL3 reporter vector digested by appropriate restriction enzymes. Primers used for the generation of mutant constructs are as follows (mutated regions are bolded and underlined CCAGGGCCCA AG**TCCCAC**CT T**CGGGGG**CGG TGCCTGGCCC as M1 and M2 respectively): M1, forward-1 (180 bp), 5′-AAA AGA TCT ACA TCT GTG TGT TGG TTT TTT GTG-3′ containing Bgl I restriction enzyme site; M1, reverse-1, 5′-GCA CCG CCC CCG AAG TTT GTC CTT GGG CCC TGG-3′; M1, forward-2 (144 bp), 5′-CCA GGG CCC AAG GAC AAA CTT CGG GGG CGG TGC CTG-3′; M1, reverse-2, 5′-AAA AAG CTT CGT CCG TGG CCA TGG TGG CCC GCG-3′ containing Hind III restriction enzyme site. Similarly, M2, forward-1 (183 bp), 5′-AAA GGT ACC ACA TCT GTG TGT TGG TTT TTT GTG-3′ containing Kpn I restriction enzyme site; M2, reverse-1, 5′-CCG GGC CAG GCA CCG TTT GTC AAG GTG GGA CTT GGG CCC-3′; M2, forward-2 (136 bp), 5′-CCC AAG TCC CAC CTT GAC AAA CGG TGC CTG GCC CGG GGA-3′; M2, reverse-2, 5′-AAA AAG CTT CGT CCG TGG CCA TGG TGG CCC GCG-3′ containing Hind III restriction enzyme site.

### Electrophoretic Mobility Shift Assay

Nuclear protein was extracted from Jurkat T cells using Nuclear and Cytoplasmic Extraction Reagents (Thermo Fisher Scientific, Waltham, MA, USA) according to the manufacturer’s instructions. For nuclear extract, 1 × 10^7^ Jurkat T cells were washed twice with ice cold PBS and incubated in 500 μl of Buffer A (10 mM HEPES, 1.5 mM MgCl2, 10 mM KCl, 0.5 mM DTT, 0.05% NP-40, pH 7.9) and 50 μl of Buffer B (5 mM HEPES, 1.5 mM MgCl_2_, 0.2 mM EDTA, 0.5 mM DTT, 26% glycerol (v/v), pH 7.9) was added to the pellets and they were vortexed roughly at 4 °C for 30 min. Biotin-labeled wild-type and competitor probes used in EMSA were shown in Table 1. After binding reactions performed between nuclear protein and each probes, the reactions were loaded into the 6% polyacrylamide gel and transferred to Biodyne B Nylon Membrane (Thermo Fisher Scientific, Waltham, MA, USA). The membranes were cross-linked, blocked, and then incubated with Stabilized Streptavidin-Horseradish Peroxidase Conjugate. Finally, membranes were incubating with chemiluminescence substrate and visualize the DNA-protein interactions.

**Table 1:** Sequences of probes.

Target probes	Probe sequences
Wild type	5′ Biotin-CCAGGGCCCAAGTCCCACCTTCGGGGGCGGTGCC-3′ Biotin
Competitor-90	5′-CCAGGGCCCAGAAACAACCTTCGGGGGCGGTGCC-3′
Competitor-88	5′-CCAGGGCCCAAGGAAACACTTCGGGGGCGGTGCC-3′
Competitor-85	5′-CCAGGGCCCAAGTCCGAAACACGGGGGCGGTGCC-3′
Competitor-82	5′-CCAGGGCCCAAGTCCCACGAAACAGGGCGGTGCC-3′
Competitor-79	5′-CCAGGGCCCAAGTCCCACCTTGAAACACGGTGCC-3′
Competitor-78	5′-CCAGGGCCCAAGTCCCACCTTCGAAACAGGTGCC-3′
Competitor-77	5′-CCAGGGCCCAAGTCCCACCTTCGGAAACAGTGCC-3′
Competitor-76	5′-CCAGGGCCCAAGTCCCACCTTCGGGAAACATGCC-3′
Competitor-73	5′-CCAGGGCCCAAGTCCCACCTTCGGGGGGAAACAC-3′
Competitor-70	5′-CCAGGGCCCAAGTCCCACCTTCGGGGGCGGGAAACA-3′

### Chromatin immunoprecipitation (ChIP) assay

Chromatin immunoprecipitation (ChIP) analysis was carried out by using the Simple ChIP Enzymatic Chromatin IP Kit (Cell Signaling Technology, Danvers, MA, USA) according to the manufacturer’s instructions. Briefly Jurkat T cells were fixed with formaldehyde to cross-link proteins to DNA. Chromatin was digested with Micrococcal Nuclease into 150–900 bp DNA/protein fragments. Swiprosin-1 antibody specific to proteins were added, and the complex co-precipitates and was captured by Protein G magnetic beads. Cross-links were reversed, and DNA was purified and ready for real-time quantitative PCR. Chromatin was immunoprecipitated with anti-Swiprosin-1 Ab (Abcam, Cambridge, UK) or rabbit IgG Ab (Cell Signaling Technology Danvers, MA, USA). Following reversal of crosslinks, presence of the selected DNA sequence was assessed by real-time qPCR using 2xSYBR green PCR mix Taq II (Tli RnaseH Plus) (RR82LR; Takara, Ann Arbor, MI, USA) by ABI 7500 (Applied Biosystems, Foster City, CA, USA). The primer sequences used in ChIP were shown in Table 2. As a loading control, the PCR was done with human RPL30 primers which primer sets are included for the human positive control RPL30 gene loci. Data are presented as the amount of DNA recovered relative to the wild type of ADR1 and Sp1.

**Table 2:** Sequences of primers.

Target Primers	Primer sequences
Wild type	Forward; 5′-CCAGGGCCCAAGTCCCACCTTCGGGGGCGGTGCC-3′
	Reverse; 5′-GGC CCG CGC GGC ACT CGC CTT-3′
Competitor-90	Forward; 5′-CCAGGGCCCAGAAACAACCTTCGGGGGCGGTGCC-3′
	Reverse; 5′-GGC CCG CGC GGC ACT CGC CTT-3′
Competitor-88	Forward; 5′-CCAGGGCCCAAGGAAACACTTCGGGGGCGGTGCC-3′
	Reverse; 5′-GGC CCG CGC GGC ACT CGC CTT-3′
Competitor-85	Forward; 5′-CCAGGGCCCAAGTCCGAAACACGGGGGCGGTGCC-3′
	Reverse; 5′-GGC CCG CGC GGC ACT CGC CTT-3′
Competitor-82	Forward; 5′-CCAGGGCCCAAGTCCCACGAAACAGGGCGGTGCC-3′
	Reverse; 5′-GGC CCG CGC GGC ACT CGC CTT-3′
Competitor-79	Forward; 5′-CCAGGGCCCAAGTCCCACCTTGAAACACGGTGCC-3′
	Reverse; 5′-GGC CCG CGC GGC ACT CGC CTT-3′
Competitor-78	Forward; 5′-CCAGGGCCCAAGTCCCACCTTCGAAACAGGTGCC-3′
	Reverse; 5′-GGC CCG CGC GGC ACT CGC CTT-3′
Competitor-77	Forward; 5′-CCAGGGCCCAAGTCCCACCTTCGGAAACAGTGCC-3′
	Reverse; 5′-GGC CCG CGC GGC ACT CGC CTT-3′
Competitor-76	Forward; 5′-CCAGGGCCCAAGTCCCACCTTCGGGAAACATGCC-3′
	Reverse; 5′-GGC CCG CGC GGC ACT CGC CTT-3′
Competitor-73	Forward; 5′-CCAGGGCCCAAGTCCCACCTTCGGGGGGAAACAC-3′

### Construction of conserved noncoding sequences

To identify a potential regulatory locus, comparative genomic analysis was performed. Genomic sequences spanning the Swiprosin-1 gene were analyzed using the Web-based alignment software VISTA browser 2.0^25^. The conserved noncoding sequences (CNS) -1, -2 and -3 constructs were generated by cloning the genomic sequences between the first and second exon of the Swiprosin-1 gene into the Sac I site located upstream of the luciferase gene in -141/Swiprosin-1 pGL3 reporter vector digested by appropriate restriction enzymes. Different combinations of forward primers containing Sac I restriction enzyme site and reverse primers containing Hind III restriction enzyme site and primers sequences as follows: CNS-1 (251 bp), forward, 5′-AAA GAG CTC GTA CTC TGA GGG CGA CTG AGG GGA-3′; reverse, 5′-AAA AAG CTT ACT TCT TCC ACG AGA GCC CGT CTG TTG-3′; CNS-2 (123 bp), forward, 5′-AAA GAG CTC CCC TCC TTC TCT CCT GCT GAA CAG-3′; reverse, 5′-AAA AAG CTT ACT TTT TTG TGC CAC CTG CCT TCC CTG-3′ and CNS-3 (143 bp), forward, 5′-AAA GAG CTC CCT CTT GGT GAT GGA CTC TTT-3′; reverse, 5′-AAA AAG CTT ACT CAA ACC ATG ATT TGA ACG GCC GCC-3′. The sequences of all the PCR derived constructs were confirmed by DNA sequencing.

### Luciferase reporter assays

Above all plasmid DNAs were prepared by DNA-spin Plasmid DNA Purification Kit (iNtRON Biotechnology, Seoul, Korea) and Jurkat T cells (1.5 × 10^6^) was transfected with 100 μl of Amaxa’s Nucleofector solution (Amaxa Biosystems, Cologne, Germany) containing 1 µg of full-length of Swiprosin-1 reporter construct or 5′ deletion mutant constructs or over-lapping PCR of M1 and M2 mutant constructs or CNS -1, -2 and -3 constructs or 1 µg of pGL3 basic vector as a control with 1 µg of pRL-TK renilla, according to the manufacturer’s protocol. Then transfected cells were immediately transferred to 2.0 ml of complete medium and cultured in 6-well plates at 37 °C. After 36 h of transfection, the medium was replaced with RPMI-1640 or IMDM medium containing 10% FBS and antibiotics. Cells were stimulated with or without PMA or A23187 or PMA/A23187 and incubated at 37 °C for 6 h. Cell lysates were prepared and assayed for luciferase activity using the Dual Luciferase Assay System (Promega, Madison, WI), according to the manufacturer’s instructions.

### Computational analysis of the Swiprosin-1 locus

To identify potential regulatory locus, comparative genomic analysis was performed. Genomic sequences spanning the Swiprosin-1 gene were analyzed using the web-based alignment software VISTA browser 2.0^32^. Transcription factor binding sites were identified using the rVISTA 2.0 software^33^, which uses matrices of the TRANSFAC database^34^.

### Statistical analysis

Statistical analyses of data were performed by using GraphPad Prism version 5. Statistical significance for the differences in the data from the control and experimental groups were determined by one-way ANOVA followed by the Newman–Keuls test and Mann-Whitney test (significance at *p, 0.05, **p, 0.01, and ***p, 0.001). Significance was only indicated when appropriate. Data are the mean of SD of at least three independent experiments, unless differently specified in the text.

## References

[1] Thylur RP, Gowda R, Mishra S, Jun CD (2018). Swiprosin-1: Its Expression and Diverse Biological Functions. J. Cell. Biochem.

[2] Thylur RP (2009). Swiprosin-1 is expressed in mast cells and up-regulated through the protein kinase C beta I/eta pathway. J. Cell. Biochem.

[3] Mielenz D, Gunn-Moore F (2016). Physiological and pathophysiological functions of Swiprosin-1/EFhd2 in the nervous system. Biochem. J.

[4] Purohit P (2014). The Ca2+ sensor protein swiprosin-1/EFhd2 is present in neurites and involved in kinesin-mediated transport in neurons. PLoS. One.

[5] Morowski M, Brachs S, Mielenz D, Nieswandt B, Dutting S (2014). The adaptor protein Swiprosin-1/EFhd2 is dispensable for platelet function in mice. PLoS. One.

[6] Wang ZB (2017). LY333531, a PKCbeta inhibitor, attenuates glomerular endothelial cell apoptosis in the early stage of mouse diabetic nephropathy via down-regulating swiprosin-1. Acta. Pharmacol. Sin.

[7] Huh YH (2015). Swiprosin-1 stimulates cancer invasion and metastasis by increasing the Rho family of GTPase signaling. Oncotarget.

[8] Grande G (2018). Semen Proteomics Reveals the Impact of Enterococcus faecalis on male Fertility. Protein. Pept. Lett.

[9] Wang RM (2018). Swiprosin-1 Promotes Mitochondria-Dependent Apoptosis of Glomerular Podocytes via P38 MAPK Pathway in Early-Stage Diabetic Nephropathy. Cell. Physiol. Biochem.

[10] Regensburger M (2018). Impact of Swiprosin-1/Efhd2 on Adult Hippocampal Neurogenesis. Stem. Cell. Reports.

[11] Kim YD (2013). Swiprosin-1 Expression Is Up-Regulated through Protein Kinase C-theta and NF-kappaB Pathway in T Cells. Immune. Netw.

[12] Avramidou A (2007). The novel adaptor protein Swiprosin-1 enhances BCR signals and contributes to BCR-induced apoptosis. Cell. Death. Differ.

[13] Ramesh TP, Kim YD, Kwon MS, Jun CD, Kim SW (2009). Swiprosin-1 Regulates Cytokine Expression of Human Mast Cell Line HMC-1 through Actin Remodeling. Immune. Netw.

[14] Kroczek C (2010). Swiprosin-1/EFhd2 controls B cell receptor signaling through the assembly of the B cell receptor, Syk, and phospholipase C gamma2 in membrane rafts. J. Immunol.

[15] Dutting S, Brachs S, Mielenz D (2011). Fraternal twins: Swiprosin-1/EFhd2 and Swiprosin-2/EFhd1, two homologous EF-hand containing calcium binding adaptor proteins with distinct functions. Cell. Commun. Signal.

[16] Hagen S (2012). The B cell receptor-induced calcium flux involves a calcium mediated positive feedback loop. Cell. Calcium.

[17] Kwon MS (2013). Swiprosin-1 is a novel actin bundling protein that regulates cell spreading and migration. PLoS. One.

[18] Brachs S (2014). Swiprosin-1/EFhd2 limits germinal center responses and humoral type 2 immunity. Eur. J. Immunol.

[19] Zhang, S. *et al*. Swiprosin-1 deficiency impairs macrophage immune response of septic mice. *JCI*. *Insight* **3**, 10.1172/jci.insight.95396 (2018). 10.1172/jci.insight.95396 PMC5821171 29415882

[20] Tu Y (2018). EFhd2/swiprosin-1 regulates LPS-induced macrophage recruitment via enhancing actin polymerization and cell migration. Int. Immunopharmacol.

[21] Nippert, F., Schreckenberg, R. & Schluter, K. D. Isolation and Cultivation of Adult Rat Cardiomyocytes. *J*. *Vis*. *Exp*, 10.3791/56634 (2017). 10.3791/56634 PMC5752423 29155786

[22] Nippert F, Schreckenberg R, Hess A, Weber M, Schluter KD (2016). The Effects of Swiprosin-1 on the Formation of Pseudopodia-Like Structures and beta-Adrenoceptor Coupling in Cultured Adult Rat Ventricular Cardiomyocytes. PLoS. One.

[23] Wang ZB (2017). Low level of swiprosin-1/EFhd2 in vestibular nuclei of spontaneously hypersensitive motion sickness mice. Sci. Rep.

[24] Vuadens F (2004). Identification of swiprosin 1 in human lymphocytes. Proteomics.

[25] Hwang JS (2015). NFAT1 and JunB cooperatively regulate IL-31 gene expression in CD4+ T cells in health and disease. J. Immunol.

[26] Komarnitsky PB, Klebanow ER, Weil PA, Denis CL (1998). ADR1-mediated transcriptional activation requires the presence of an intact TFIID complex. Mol. Cell. Biol.

[27] Wang HB (2013). Sp1 and c-Myc regulate transcription of BMI1 in nasopharyngeal carcinoma. FEBS. J.

[28] Gibson HM (2007). Induction of the CTLA-4 gene in human lymphocytes is dependent on NFAT binding the proximal promoter. J. Immunol.

[29] Pahl HL (1999). Activators and target genes of Rel/NF-kappaB transcription factors. Oncogene.

[30] Loots GG (2000). Identification of a coordinate regulator of interleukins 4, 13, and 5 by cross-species sequence comparisons. Science.

[31] Strempel JM, Vercelli D (2007). Functional dissection identifies a conserved noncoding sequence-1 core that mediates IL13 and IL4 transcriptional enhancement. J. Biol. Chem.

[32] Mayor C (2000). VISTA: visualizing global DNA sequence alignments of arbitrary length. Bioinformatics.

[33] Loots GG, Ovcharenko I (2004). rVISTA 2.0: evolutionary analysis of transcription factor binding sites. Nucleic. Acids. Res.

[34] Wingender E (2001). The TRANSFAC system on gene expression regulation. Nucleic. Acids. Res.

